# Thermo-Electro-Mechanical Simulation of Electro-Active Composites

**DOI:** 10.3390/ma15030783

**Published:** 2022-01-20

**Authors:** Anas Kanan, Aleksandr Vasilev, Cornelia Breitkopf, Michael Kaliske

**Affiliations:** 1 Institute for Structural Analysis, Technische Universität Dresden, 01062 Dresden, Germany; anas_m_a.kanan@tu-dresden.de; 2 Chair of Technical Thermodynamics, Technische Universität Dresden, 01062 Dresden, Germany; aleksandr.vasilev@tu-dresden.de (A.V.); cornelia.breitkopf@tu-dresden.de (C.B.)

**Keywords:** finite element method, thermo-electro-mechanics, electro-active polymers, composite materials, molecular dynamics simulations, silicone rubber

## Abstract

In this contribution, a computational thermo-electro-mechanical framework is considered, to simulate coupling between the mechanical, electrical and thermal fields, in nonhomogeneous electro-active materials. A thermo-electro-mechanical material model and a mixed Q1P0 finite element framework are described and used for the simulations. Finite element simulations of the response of heterogeneous structures consisting of a soft matrix and a stiff incluison are considered. The behavior of the composite material is studied for varying initial temperatures, different volume fractions and various aspect ratios of the inclusion. For some of the examples, the response of the structure beyond a limit point of electro-mechanical instability is traced. Regarding the soft matrix of the composite, thermal properties of silicone rubber at normal conditions have been obtained by molecular dynamics (MD) simulations. The material parameters obtained by MD simulations are used within the finite element simulations.

## 1. Introduction

Electro-active polymers (EAP) are smart materials that can be favorably used in artificial muscles and soft robotics. That is due to EAP’s relatively quasi-instantaneous mechanical actuation in response to an applied electric field [1]. Furthermore, EAP have demonstrated a capability of exhibiting relatively large strains, where in specific types of EAP, strains up to more than 300% are recorded [2]. Many prototypes of soft robotics have been mainly based on EAP, see for instance [3,4,5]. Dielectric elastomer actuator (DEA) is a class of EAP, which demonstrates electrostrictive behavior [6]. Several researchers have investigated the influence of filling DEA’s soft matrices with stiff particles that possess relatively large electric permittivity [7,8,9,10]. It has been demonstrated that the intensity of electro-mechanical coupling in particle-filled DEA is enhanced as the volume fraction of embedded particles increases [8,9]. The influence of embedding stiff fillers within soft DEA has been numerically investigated in [10,11,12,13].

During the last century, several contributions have been devoted to the mathematical description of polarization and electro-elastic coupling [14,15,16,17]. Thereafter, more recent works are proposed to derive mathematical expressions describing different forms of electro-elastic interactions [18,19,20,21,22].

Computational modeling of electro-mechanical interactions in EAP relies either on a purely energy-based approach or, alternatively, on the relatively computationally efficient mixed energy-enthalpy-based approach [12]. In the context of the finite element method, the energy-based approach requires setting a vector electric potential (three degrees of freedom) to describe the electrical part of the problem. The mixed energy-enthalpy approach depends on a scalar electric potential (one degree of freedom) to express the electrical sub-problem. As an example of numerical solutions of the associated problem, a finite element implementation of electro-elasticity at large deformations has been implemented in [23]. Numerical investigations of composites composed of dielectric soft matrix and stiff inclusions have been performed in [12,24]. It has been shown that through driving the simulation using surface charges, the response of EAP beyond the occurrence of electro-mechanical instability can be simulated using the more convenient mixed energy-enthalpy-based approach [12]. A mixed finite element formulation for composites consisting of quasi-incompressible soft matrix and stiff fibers has been proposed in [25,26].

The actuation behavior of EAP is mainly based on coupling between the electrical field and the mechanical field. Nevertheless, temperature changes resulting due to self-induced heating or cooling, and varying ambient conditions can influence EAP’s properties and its actuation response. Therefore, the simulation of interactions between thermal, electrical and mechanical fields have been previously considered, through proposing a thermo-electro-mechanical model [27] and a thermo-electro-mechanical finite element framework [28,29]. The finite element formulation developed in [28] is a standard displacement-based formulation, and it relies on a partially-staggered solution approach.

In this work, finite element modeling of thermo-electro-mechanical interactions in nonhomogeneous electro-active materials is considered. Regarding the constitutive model used in the present work, a material description that takes into account a quasi-linear dielectric response [25] and an entropic thermo-elastic coupling [30] is adopted. A mixed Q1P0 thermo-electro-mechanical framework is implemented and used for the simulations. Regarding the estimation of thermal properties, thermal conductivity, heat capacity and coefficient of thermal expansion of silicone rubber at normal conditions, are estimated using molecular dynamics (MD) simulations, which is an alternative approach to experimental investigations for the prediction of material properties. In this work, the reason of using MD simulations is to avoid conducting time consuming experiments and to demonstrate an efficient coupling of various numerical approaches, where both MD and finite element simulations are performed to carry out the associated study. Numerical simulations of heterogeneous structures with spherical inclusions under varying initial temperature is performed. The effect of varying volume fraction and changing aspect ratio of a stiff inclusion on the overall composite’s response are numerically investigated. Both polarization behavior and intensity of electro-mechanical coupling are evaluated. All simulation examples are driven by applying surface charges. For some of the performed analyses, the structural response beyond the point of electro-mechanical instability is traced.

The present paper is organized as follows. Section 2 introduces the constitutive modeling approach and the associated finite element implementation. In Section 3, the method used to determine thermal properties of silicone rubber is described. In Section 4, several numerical examples are demonstrated. Section 5 ends the paper with a brief summary.

## 2. Thermo-Electro-Mechanics

This section is devoted to present a constitutive formulation and its associated numerical treatment, which are used to emulate thermo-electro-mechanical interactions at large deformations. Principles of continuum mechanics are demonstrated and employed within a coupled thermo-electro-mechanical environment. An overview of the kinematics and balance laws of the coupled problem is outlined. Subsequently, a constitutive model is presented. Finally, the used finite element formulation is described.

### 2.1. Preliminaries

The nonlinear deformation map x=(φ,t) at time t∈T projects points ***X*** in the reference configuration $B_{0}$ to their counterparts ***x*** in the current setting $B_{t}$. The deformation gradient $F=\nabla_{x\varphi }$, its cofactor $\mathrm{cof}\left[F\right]=det\left[F\right]F^{-T}$ and its Jacobian J=det[F] project an infinitesimal line element, area element and volume element from the reference setting to the current setting, respectively. The reference density $\rho_{0}$ is connected to the current density ρ by $\rho_{0}=J\rho$. Furthermore, a scalar electric potential ϕ(x,t) and an absolute temperature θ(x,t) are defined. The outer boundary $\partial B_{t}$ is decomposed in terms of the mechanical, electrical and thermal boundary conditions as

(1) $$\begin{array}{c} \partial B_{t}=\partial B_{t}^{\varphi }\cup \partial B_{t}^{t}\;\mathrm{with}\;\partial B_{t}^{\varphi }\cap \partial B_{t}^{t}\;=\emptyset ,\\ \partial B_{t}=\partial B_{t}^{\phi }\cup \partial B_{t}^{w}\;\mathrm{with}\;\partial B_{t}^{\phi }\cap \partial B_{t}^{w}=\emptyset ,\\ \partial B_{t}=\partial B_{t}^{\theta }\cup \partial B_{t}^{q_{\theta }}\;\mathrm{with}\;\partial B_{t}^{\theta }\cap \partial B_{t}^{q_{\theta }}=\emptyset , \end{array}$$

respectively. $\partial B_{t}^{\varphi }$, $\partial B_{t}^{\phi }$ and $\partial B_{t}^{\theta }$ are the surfaces where Dirichlet mechanical, electric and thermal boundary conditions can be prescribed, respectively. The Neumann boundaries, where mechanical tractions ***t***, surface charge densities w and surface heat flow $q_{\theta }$ can be prescribed, read $\partial B_{t}^{t}$, $\partial B_{t}^{w}$ and $\partial B_{t}^{q_{\theta }}$, respectively.

### 2.2. Mechanical Field

The right Cauchy-Green deformation tensor is introduced as

(2) $$C=F^{T}gF,$$

where ***g*** is the covariant Eulerian metric tensor. The strong form of the balance of linear momentum for quasi-static problems can be defined as

(3) $$\begin{array}{cc} \nabla_{x}\cdot \sigma +\rho \gamma^{mec} & =0\;\mathrm{in}\;B_{t} \end{array}$$

with the divergence with respect to the spatial coordinates $\nabla_{x}\cdot \left[\;\cdot \;\right]$, the total Cauchy stresses ***σ*** and the mechanical body forces per deformed unit volume $\rho \gamma^{mec}$. The total stresses can be split into mechanical and ponderomotive stresses as $\sigma =\sigma^{mec}+\sigma^{pon}$. The ponderomotive stresses $\sigma^{pon}$ express electro-mechanical coupling in the material. For further details, the reader is referred to [20,31,32].

### 2.3. Electrical Field

In the case of electro-statics, where magnetic fields are absent, the electric field at the reference configuration E and the electric field at the current configuration e are defined as

(4) $$\;E=-\nabla_{X}\phi \left(X,t\right),\;e=-\nabla_{x}\phi \left(x,t\right)=F^{-T}E,$$

where $\nabla_{X}\left[\;\cdot \;\right]$ denotes the differential operator with respect to the reference coordinates ***X***, and $\nabla_{X}\left[\;\cdot \;\right]$ is its counterpart with respect to the current coordinates ***x***. Gauss’s law for electricity can be written with its differential form in terms of the current electric displacement d as

(5) $$\nabla_{x}\cdot d=\varrho^{f}\;\mathrm{in}\;B_{t},$$

where $\varrho^{f}$ is the free charges per deformed unit volume. In insulators, there are no free volume charges ($\varrho^{f}=0)$. The electric displacement in the deformed setting d is connected to its counterpart in the undeformed setting D by

(6) $$d=J^{-1}FD.$$

The surface charge densities at the undeformed and at the deformed setting are defined as

(7) W=−D·N,w=−d·n

with the unit normal vector ***N*** on the surface $\partial B_{0}$ and the unit normal vector ***n*** on the surface $\partial B_{t}$.

### 2.4. Thermal Field

The heat flux at the reference configuration ***Q*** and its counterpart at the current configuration ***q*** are introduced as

(8) $$Q=-kC^{-1}\cdot \nabla_{X}\theta \left(X,t\right),\;q=-J^{-1}k\;\nabla_{x}\theta \left(x,t\right),$$

respectively, where *k* is a parameter of heat conductivity. The evolution of temperature is expressed by the transient heat equation, which reads

(9) $$\rho c\;\dot{\theta }+\nabla_{x}\cdot q-\rho r-\rho w_{ext}=0\;\mathrm{in}\;B_{t},$$

where ρc is the heat capacity, ρr is the heat source, $\rho w_{ext}$ is the external power, all introduced per unit deformed volume. The term of external power $\rho w_{ext}$ can be decomposed into mechanical, electro-mechanical and electrical parts as

(10) $$\begin{array}{c} \rho w_{ext}=\rho w_{ext}^{mec}+\rho w_{ext}^{coup}+\rho w_{ext}^{elec},\\ \rho w_{ext}^{mec}=\theta \partial_{\theta }\left[\sigma^{mec}:d\right],\\ \rho w_{ext}^{coup}=\theta \partial_{\theta }\left[\sigma^{pon}:d\right],\\ \rho w_{ext}^{elec}=-\theta \partial_{\theta }\left[d\cdot \left(\dot{e}+l^{T}e\right)\right], \end{array}$$

where $l=\dot{F}F^{-1}$ is the spatial velocity gradient, $d=(l+l^{T})/2$ is its symmetric part and $\dot{e}$ denotes the rate of change of the current electric field with respect to time. The terms $\rho w_{ext}^{mec}$, $\rho w_{ext}^{coup}$ and $\rho w_{ext}^{elec}$ express the work done by the mechanical stresses $\sigma^{mec}$, the ponderomotive stresses $\sigma^{pon}$ and the electric displacement d, respectively.

### 2.5. Material Model

The deformation gradient is multiplicatively decomposed into purely volumetric and isochoric (volume-preserving) contributions as $F=F^{vol}\bar{F}$, where the volumetric contribution is defined by $F^{vol}=J^{1/3}1$ and the isochoric contribution is given by $\bar{F}=J^{-1/3}F$. The isochoric part of the right Cauchy-Green deformation tensor is introduced as $\bar{C}={\bar{F}}^{T}g\bar{F}$. Electro-mechanical and entropic thermo-mechanical couplings of the material are expressed through the energy density function

(11) $$\begin{array}{cc} \Psi^{tot}\left(J,C,E,\theta \right) & =\frac{\theta }{\theta_{0}}\;\Psi_{e}^{vol}\left(J\right)+\frac{\theta }{\theta_{0}}\;\Psi_{e}^{iso}\left(\bar{C}\right)+\left(1-\frac{\theta }{\theta_{0}}\right)e_{0}\left(J\right)\\ & +T_{0}\left(\theta \right)+\Psi^{coup}\left(\bar{C},E\right), \end{array}$$

where $\theta_{0}$ is the reference temperature. The volumetric contribution of the mechanical energy density is introduced as

(12) $$\Psi_{e}^{vol}\left(J\right)=\kappa \left(J-\ln J-1\right),$$

where κ is the bulk modulus. The isochoric part $\Psi_{e}^{iso}$ of the mechanical energy function is specified by the Neo-Hookean model as

(13) $$\Psi_{e}^{iso}\left(\bar{C}\right)=\frac{1}{2}G_{c}\left({\bar{I}}_{\bar{C}}-3\right),$$

where $G_{c}$ is the shear modulus and ${\bar{I}}_{\bar{C}}=\mathrm{tr}\bar{C}$ denotes the first invariant of the isochoric right Cauchy-Green tensor. The thermal expansion is expressed in terms of the reference internal energy

(14) $$e_{0}\left(J\right)=\kappa \alpha \theta_{0}\ln J$$

with the coefficient of thermal expansion α [30]. The thermal function $T_{0}\left(\theta \right)$ is introduced by

(15) $$T_{0}\left(\theta \right)=\rho_{0}c\left(\theta -\theta_{0}-\theta \ln\left(\frac{\theta }{\theta_{0}}\right)\right),$$

where $\rho_{0}c=-\theta \partial_{\theta \theta }^{2}T_{0}\left(\theta \right)$ denotes the heat capacity per unit reference volume. The coupled electro-mechanical contribution of the energy function $\Psi^{coup}$ is considered as it is suggested in [12,25], and it is introduced here as

(16) $$\Psi^{coup}\left(\bar{C},E\right)=-\frac{1}{2}\epsilon_{r}\epsilon_{0}\;{\bar{C}}^{-1}:\left[E⊗E\right],$$

where $\epsilon_{r}$[−] denotes the relative electric permittivity of the material and $\epsilon_{0}=8.854\times 10^{-12}\;{N/V}^{2}$ is the electric permittivity of vacuum. The total Cauchy stresses ***σ*** and the ponderomotive stresses $\sigma^{pon}$ can be computed by

(17) $$\sigma =2J^{-1}\partial_{g}\Psi^{tot}\left(J,C,E,\theta \right),\;\sigma^{pon}=2J^{-1}\partial_{g}\Psi^{coup}\left(J,C,E\right),$$

respectively. The current electric displacement d is introduced as

(18) $$d=-J^{-1}\partial_{e}\Psi^{coup}\left(\bar{C},E\right).$$

As it is shown in Equation (11), the electro-mechanical contribution $\Psi^{coup}\left(J,C,E\right)$ is not scaled with a temperature field. Therefore, the the ponderomotive stresses $\sigma^{pon}$ and the electric displacement d as defined in Equations (17) and (18), respectively, are temperature independent. Due to that, the external power terms $\rho w_{ext}^{coup}$ and $\rho w_{ext}^{elec}$, as defined in Equation (10), are equal to zero.

### 2.6. Finite Element Formulation

In order to avoid volumetric locking that can arise due to the material’s nearly incompressible behavior, a mixed Q1P0 formulation is adopted and implemented within a thermo-electro-mechanical framework. To this end, the mean dilatation $D^{e}$ and the mean pressure $p^{e}$ are introduced as additional field variables and treated as averages on finite element level [25,33]. The method of weighted residuals as previously used in [24,25,30], is employed to formulate a thermo-electro-mechanical finite element framework. To this end, the main strong forms as given in Equations (3), (5) and (9) are scaled with the test functions $\delta_{\varphi }$, δϕ and δθ, respectively. Thereafter, integration by parts is applied and the Gauss theorem is used. Performing the latter two steps allows to express the associated weak forms as

(19) $$\begin{array}{cc} G^{\varphi }\left(\delta_{\varphi },\varphi ,\phi ,\theta ,p^{e}\right) & =\int_{B_{t}}\nabla_{x}\delta_{\varphi }:\sigma \;dv-\int_{\partial B_{t}}\delta_{\varphi }\cdot t\;da-\int_{B_{t}}\delta_{\varphi }\cdot \rho \gamma^{mec}\;dv=0,\\ G^{\phi }\left(\delta \phi ,\phi ,\varphi ,\theta \right) & =\int_{B_{t}}\nabla_{x}\delta \phi \cdot d\;dv+\int_{\partial B_{t}}\delta \phi \;w\;da+\int_{B_{t}}\delta \phi \;\varrho^{f}\;dv=0,\\ G^{\theta }\left(\delta \theta ,\theta ,\varphi ,\phi ,p^{e}\right) & =\int_{B_{t}}\delta \theta \;\rho c\;\dot{\theta }\;dv-\int_{B_{t}}\nabla_{x}\delta \theta \cdot q\;dv+\int_{\partial B_{t}}\delta \theta \;q_{\theta }\;da\\ & -\int_{B_{t}}\delta \theta \;\rho r\;dv-\int_{B_{t}}\delta \theta \;\rho w_{ext}\;dv=0. \end{array}$$

In Equation (19), the surface tractions ***t***, the surface charge density w and the surface heat flow $q_{\theta }$ are introduced as t=σ·n on $\partial B_{t}^{t}$, w=−d·n on $\partial B_{t}^{w}$ and $q_{\theta }=q\cdot n$ on $\partial B_{t}^{q_{\theta }}$, respectively. The finite element implementation of the problem is performed through discretizing the weak forms in Equation (19) and the associated incremental forms. Therefore, the undeformed domain $B_{0}$ is split into finite elements as $B_{0}\approx {⋃}_{e=1}^{n_{e}}B_{0}^{e}$, where $n_{e}$ is the total number of solid elements. To treat the material model within the mixed Q1P0 finite element framework, the total energy density function as given by Equation (11) is additively decomposed into two contributions and is reformulated to

(20) $$\Psi^{tot}\left(D^{e},\bar{C},E,\theta \right)=\hat{\Psi }\left(\bar{C},E,\theta \right)+\Psi_{tot}^{vol}\left(D^{e},\theta \right),$$

where $\hat{\Psi }$ is considered on each Gauss point. The volumetric part $\Psi_{tot}^{vol}$ is parameterized in terms of the mean dilatation $D^{e}$ and can be written as

(21) $$\Psi_{tot}^{vol}\left(D^{e},\theta \right)=\frac{\theta }{\theta_{0}}\;\Psi_{e}^{vol}\left(D^{e}\right)+\left(1-\frac{\theta }{\theta_{0}}\right)e_{0}\left(D^{e}\right).$$

The average dilatation and average mean pressure are evaluated over each finite element as

(22) $$D^{e}=\frac{1}{V^{e}}\int_{B_{0}^{e}}J\;dV\;\mathrm{and}\;p^{e}=\partial_{D^{e}}\Psi_{tot}^{vol}\left(D^{e},\theta \right).$$

In Equation (19), the total Cauchy stresses are additively decomposed as $\sigma =\hat{\sigma }+\sigma^{vol}$, where the part $\hat{\sigma }=2J^{-1}\partial_{g}\hat{\Psi }$ is computed at Gauss points and the volumetric contribution $\sigma^{vol}=p^{e}g^{-1}$ is evaluated at each element $B_{t}^{e}$. The method followed to treat the power term $\rho w_{ext}$ within the Q1P0 finite element is detailed in [33]. The isoparametric concept of the finite element method is utilized, where the fields $\varphi^{e}$, $\phi^{e}$, $\theta^{e}$ and the associated test functions are interpolated over the finite element $B_{t}^{e}$ using nodal values and Lagrangian shape functions. Regarding the solution scheme adopted, the thermo-electro-mechanical problem is decomposed into an electro-mechanical and a thermal sub-problem. The electro-mechanical part of the problem is solved monolithically at a fixed temperature. Thereafter, the thermal sub-problem is solved and attached to the whole system using a staggered transfer of solution fields [28]. The evaluation of both sub-problems is based on an exchange of information after each iteration. The presented material model and finite element formulation are implemented within an in-house finite element program.

## 3. Estimation of Thermal Properties

**Figure 1:**
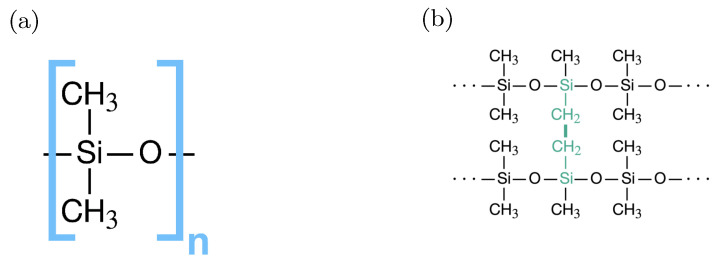
(**a**) Monomer unit of silicone; (**b**) crosslink bridge between silicone chains.

**Figure 2:**
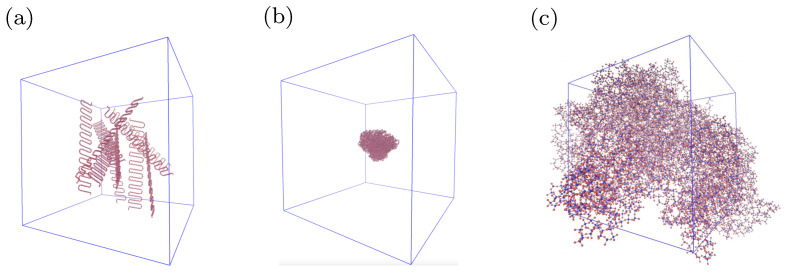
(**a**) Randomly distributed silicone chains in a periodic supercell; (**b**) the silicone chains after crosslinking procedure; (**c**) model of silicone rubber.

Firstly, polymeric chains, which consist of 418 monomer units, have been constructed by the Moltemplate software [34]. The monomer unit of silicone rubber is shown in Figure 1a. After that, ten polymeric chains have been randomly distributed in a periodic supercell (see Figure 2a) by Packmol software [35]. In the next step, the chains have been crosslinked in a canonical ensemble via an algorithm similar to the one presented in [36]. During this procedure, CH${}_{3}$ groups participating in the creation of a bond between the polymeric chains are turned into CH${}_{2}$ groups. The crosslink bridge of silicone rubber is shown in Figure 1b. The degree of crosslinking of the obtained model of silicone rubber is equal to 9.8 %. It is defined as given in [37].

The force field description has been used to describe interactions between the atoms, where the CH${}_{3}$ group has been modeled as one united atom. The total potential energy of a polymeric system is calculated in this approximation as

(23) $$E=E_{\mathrm{bond}}+E_{\mathrm{angle}}+E_{\mathrm{dihedral}}+E_{\mathrm{non}-\mathrm{bonded}}.$$

Non-bonded interactions are modeled only with van der Waals interactions. The cutoff distance is set to 10 Å in all simulations. The Lorentz-Berthelot mixing rules are used to find the missing parameters of the Lennard-Jones potential. The force field parameters have been taken from the OPLS-AA (All Atom) [38] force field, except the parameters of Lennard-Jones potential for the CH${}_{3}$ group, which has been modeled by the OPLS-UA (United Atom) [38] force field. All simulations are carried out using the LAMMPS [39] software package.

After the crosslinking procedure, density of the system has been equilibrated in an isothermal-isobaric ensemble at normal conditions for 200 ps. Before calculation of thermal conductivity by the Green-Kubo method, it has been modeled in a canonical ensemble for 100 ps for equilibration of temperature. By the Green-Kubo formula, the thermal conductivity of an isotropic material can be found as

(24) $$\lambda =\frac{V}{3k_{B}T^{2}}\int_{0}^{\infty }\;h\left(0\right)h\left(t\right)>dt,$$

where *h* is the heat flux calculated as

(25) $$h=\frac{1}{V}\left[\;\sum_{i}e_{i}\vec{\upsilon_{i}}-\sum_{i}S_{i}\vec{\upsilon_{i}}\;\right],$$

where $e_{i}$ is the total energy of *i*-th atom, $S_{i}$ is the stress tensor of *i*-th atom, $\vec{\upsilon_{i}}$ and $\vec{\upsilon_{j}}$ are velocities of *i*-th and *j*-th atoms.

The model of silicone rubber has been simulated in a microcanonical ensemble for 3 ns with a time step of 1 fs. The heat flux has been calculated at each time step and the thermal conductivity has been obtained for each correlation time interval, which is equal to 3 ps.

For calculation of specific heat capacity at constant pressure and coefficient of thermal expansion, the model has been simulated in an isothermal-isobaric ensemble at normal conditions for 100 ps and data points at equilibrium have been taken. The specific heat capacity and the coefficient of thermal expansion have been obtained as derivatives of enthalpy and volume as in [40,41,42]

(26) $$c_{p}=\frac{H(p,T+\Delta T)-H(p,T-\Delta T)}{2\Delta T},$$

(27) $$\alpha =\frac{1}{V(p,t)}\frac{V(p,T+\Delta T)-V(p,T-\Delta T)}{2\Delta T}\cdot$$

**Figure 3:**
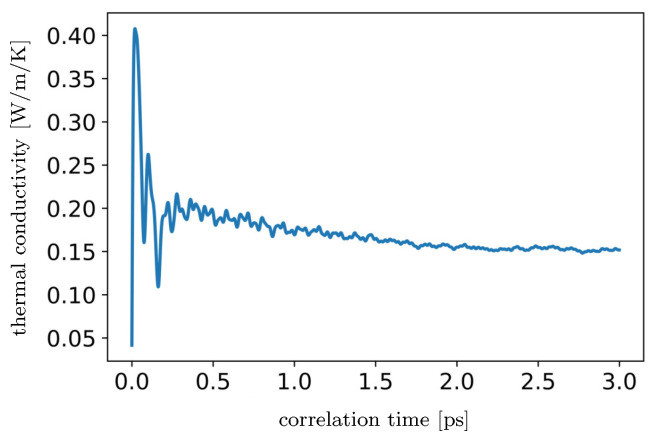
Thermal conductivity of silicone rubber at normal conditions as function of correlation time in picosecond.

The thermal conductivity obtained by the Green-Kubo method is roughly 0.15 W/m/K (see Figure 3), which is close to experimental value (≈0.17 W/m/K [43]). The specific heat capacity at constant pressure found by the MD simulations is around 1500 J/kg/K. For comparison, in [44], it is approximately 1.2 kJ/kg/K. Calculated coefficient of thermal expansion (2.83 × $10^{-4}$ 1/K) is close to measured value (roughly 3 $\times \;10^{-4}$ 1/K [45]).

## 4. Numerical Examples

**Figure 4:**
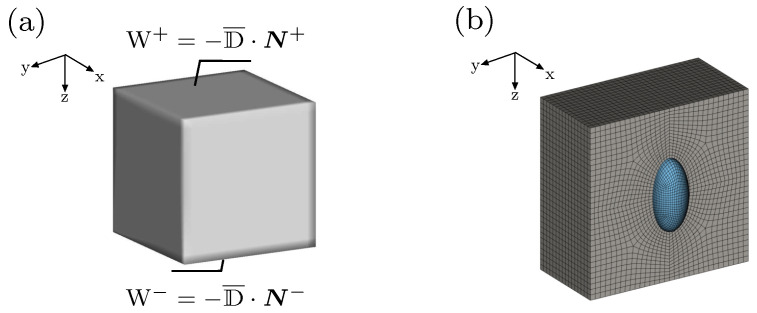
(**a**) Schematic representation of the structure with applied surface charge density W, (**b**) finite element mesh of the structure showing one-half of the soft matrix and the full inclusion with a volume fraction f=1% and an aspect ratio r=2[−].

This section is devoted to present several numerical studies of the coupled thermo-electro-mechanical response of heterogeneous structures using the finite element meth-od. An electro-active composite consisting of soft matrix and stiff inclusion is considered. The material parameters are taken as they are shown in Table 1. An initial Poisson’s ratio $\nu_{0}=0.499$ is considered. The simulations are driven by applying electrical Neumann boundary conditions, where surface charges are prescribed on the top and the bottom surfaces of the composite, as demonstrated in Figure 4a. That permits to trace the structural response beyond an electro-mechanical instability point. The surface charge density W is defined in terms of volume-averaged electric displacement $\bar{D}$, as indicated in Figure 4a. Mechanical Dirichlet boundary conditions are assigned to mid-planes of the structure, where the motion along each mid-plane’s perpendicular direction is constrained. Similar electrical and mechanical boundary conditions have been previously used in [12]. As the geometry and the boundary conditions are symmetric, only one-eighth of the whole composite is analyzed. All considered configurations are discretized using eight-noded Q1P0 finite elements. The discretization of a structure consisting of soft matrix and ellipsoidal stiff inclusion is shown in Figure 4b. In the first example, the effect of varying initial temperature on the response of a composite with spherical inclusion is studied. The second example is meant to examine the influence of varying volume fraction of the inclusion on the composite’s response. In the third example, heterogeneous structures with different aspect ratios of the inclusion are studied.

The polarization behavior is evaluated through examining the relation between the electric field and the electric displacement. Moreover, the intensity of electro-mechanical coupling is investigated in terms of the relation between the electric field and the deformation. The global response of the composites is evaluated using normalized and averaged electric field $\tilde{\bar{E}}$[−], normalized and averaged electric displacement $\tilde{\bar{D}}$[−], and averaged deformation gradient $\bar{F}$, which are defined by

(28) $$\tilde{\bar{E}}=\frac{1}{V\sqrt{G_{c}/\left(\epsilon_{r}\epsilon_{0}\right)}}\int_{B_{0}}E\;dV,\;\tilde{\bar{D}}=\frac{1}{V\sqrt{G_{c}\epsilon_{r}\epsilon_{0}}}\int_{B_{0}}D\;dV,\;\bar{F}=\frac{1}{V}\int_{B_{0}}F\;dV,$$

where here $\epsilon_{r}$ denotes the relative electric permittivity of the soft matrix, $G_{c}$ is the shear modulus of the soft matrix and *V* is the total volume of the structure.

### 4.1. Varying Initial Temperature

**Figure 5:**
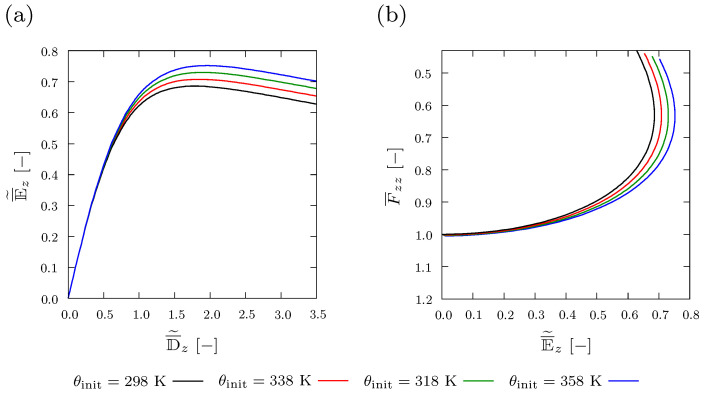
Plot of (**a**) averaged and normalized electric displacement ${\tilde{\bar{D}}}_{z}$[−] and (**b**) averaged deformation ${\bar{F}}_{zz}$[−] versus averaged and normalized electric field ${\tilde{\bar{E}}}_{z}$[−] with varying applied initial temperature $\theta_{\mathrm{init}}=\left\{298,\;318,\;338\;\mathrm{and}\;358\;\mathrm{Kelvin}\right\}$.

The influence of varying initial temperature $\theta_{\mathrm{init}}$ on the electro-mechanical coupling and the polarization behavior of a composite, where entropic thermo-elastic coupling takes place, is investigated. The structure considered here has a spherical inclusion with volume fraction f=1%. The polarization behavior is represented by the relation between the dimensionless electric displacement ${\tilde{\bar{D}}}_{z}$[−] and the dimensionless electric field ${\tilde{\bar{E}}}_{z}$[−], both evaluated along *z*-direction. Figure 5a shows that as the temperature increases, lower values of electric field ${\tilde{\bar{E}}}_{z}$[−] result in response to the same applied electric displacement ${\tilde{\bar{D}}}_{z}$[−]. Electro-mechanical coupling is evaluated using the relation between ${\tilde{\bar{E}}}_{z}$[−] and the averaged stretch in *z*-direction ${\bar{F}}_{zz}$[−]. Figure 5b demonstrates that increasing the initial temperature leads to less deformation of the composite, in response to the same electric field.

**Figure 6:**
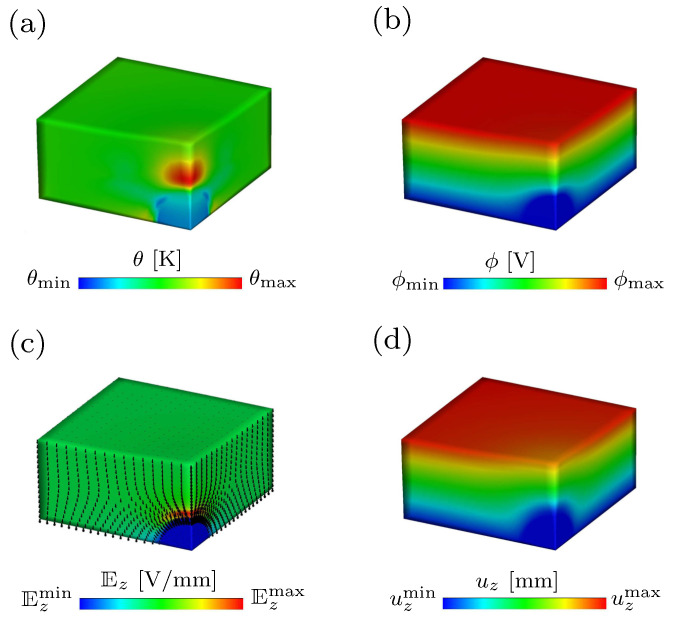
Finite element simulation of the heterogeneous structure at ${\tilde{\bar{D}}}_{z}=1.75$[−] and $\theta_{\mathrm{init}}=298$ Kelvin, showing the contour map of (**a**) absolute temperature θ in Kelvin, (**b**) electric potential ϕ in Volt, (**c**) electric field $E_{z}$ in Volt per millimeter and (**d**) displacement $u_{z}$ in millimeter.

The temperature distribution within the composite at an applied electric displacement ${\tilde{\bar{D}}}_{z}=1.75$[−] is shown in Figure 6a. The distribution of the electric potential ϕ is depicted in Figure 6b, where it can be noticed that there is a discontinuity in the distribution of ϕ. Figure 6c demonstrates that the electric fields within the inclusion tend to zero, compared to the fields within the region of soft matrix. That is due to the inclusion’s relatively high electric permittivity. The vectors in Figure 6c indicate the direction of the electric field at the reference configuration $E_{z}$. Figure 6d shows the distribution of the vertical displacement within both, the soft matrix with relatively low shear modulus and the stiff inclusion with relatively high shear modulus.

### 4.2. Varying Volume Fraction of the Inclusion

**Figure 7:**
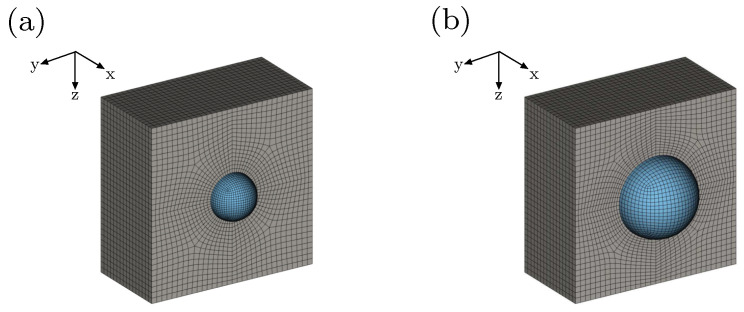
Finite element mesh of one half of the soft matrix and the whole spherical inclusion with a volume fraction (**a**) f=1% and (**b**) f=5%.

**Figure 8:**
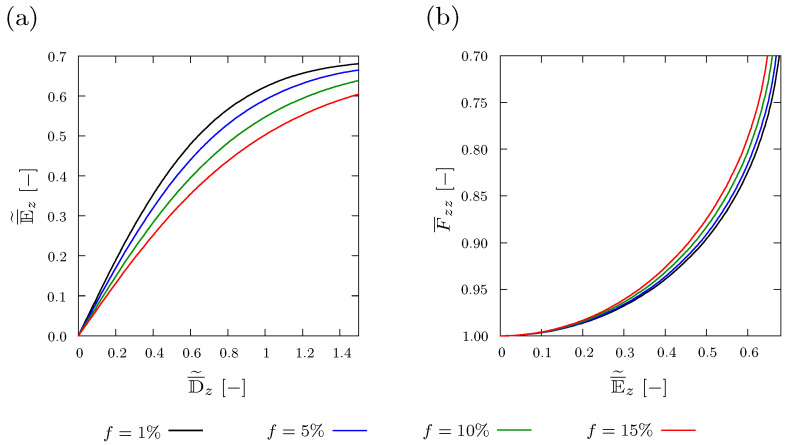
Plot of (**a**) averaged and normalized electric displacement ${\tilde{\bar{D}}}_{z}$[−] and (**b**) averaged deformation ${\bar{F}}_{zz}$[−] versus averaged and normalized electric field ${\tilde{\bar{E}}}_{z}$[−] with different volume fractions *f* of a spherical inclusion with $\theta_{\mathrm{init}}=298$ Kelvin.

The influence of different volume fractions f={1%,5%,10%and15%} of a spherical inclusion with respect to the whole composite, is studied in this section. Figure 7a,b demonstrate finite element discretizations of a composite with f=1% and a composite with f=5%, respectively. The results show that increasing the volume fraction *f* leads to higher polarization intensity in terms of ${\tilde{\bar{D}}}_{z}$[−] for the same value of the electric field ${\tilde{\bar{E}}}_{z}$[−], as demonstrated in Figure 8a. Regarding electro-mechanical coupling, Figure 8b shows that the structure deforms more as the volume fraction of the stiff inclusion *f* increases under the same electric field ${\tilde{\bar{E}}}_{z}$[−].

### 4.3. Varying Aspect Ratio of the Inclusion

**Figure 9:**
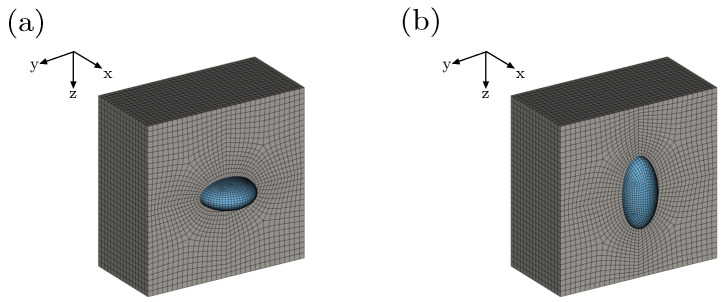
Finite element mesh of one half of the soft matrix and the whole inclusion with aspect ratio (**a**) r=0.5[−] and (**b**) r=2.0[−], where f=1%.

The effect of varying aspect ratio of the inclusion r={0.5[−],1[−]and2[−]} on the global response of the composite, is studied. Finite element meshes of composites with r=0.5[−] and r=2[−] are depicted in Figure 9a,b, respectively.

**Figure 10:**
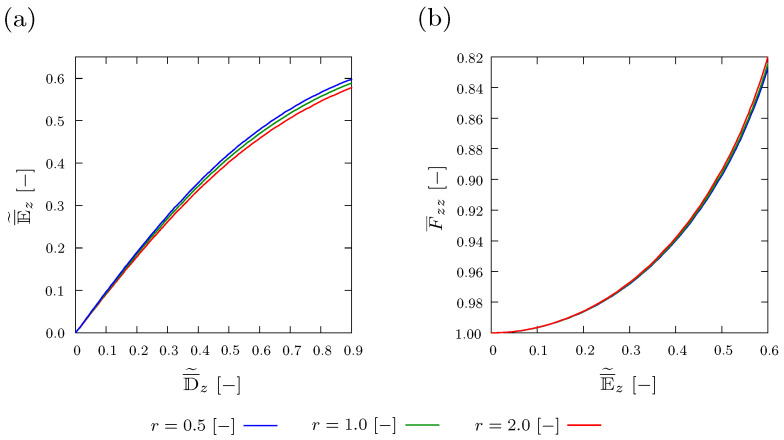
Plot of (**a**) averaged and normalized electric displacement ${\tilde{\bar{D}}}_{z}$[−] and (**b**) averaged deformation ${\bar{F}}_{zz}$[−] versus averaged and normalized electric field ${\tilde{\bar{E}}}_{z}$[−] with different aspect ratios *r*[−] of the inclusion, where f=1% and $\theta_{\mathrm{init}}=298$ Kelvin.

Figure 10a shows that as the aspect ratio *r*[−] increases, lower values of ${\tilde{\bar{E}}}_{z}$[−] correspond to the same applied ${\tilde{\bar{D}}}_{z}$[−]. The results in Figure 10a demonstrate that the influence of *r* on the ${\tilde{\bar{D}}}_{z}-{\tilde{\bar{E}}}_{z}$ relation is relatively insignificant. Figure 10b depicts the influence of *r* on the relation between ${\tilde{\bar{E}}}_{z}$[−] and ${\bar{F}}_{zz}$[−]. It is noticed from Figure 10b that for the same electric field ${\tilde{\bar{E}}}_{z}$[−], the deformation in z− direction increases slightly by increasing *r*.

## 5. Summary

In the present work, finite element analyzes of thermo-electro-mechanical interactions in heterogeneous structures are performed. The considered structures are composed of a soft matrix and a stiff inclusion. The influences of varying initial temperatures, different volume fractions and varying aspect ratios of the stiff inclusion on the overall response of the composite are studied. For the evaluation of the material’s response, both the polarization and the electro-mechanical coupling are evaluated. The thermal material parameters of the soft matrix, namely, thermal conductivity, heat capacity and coefficient of thermal expansion are identified based on molecular dynamics (MD) simulations of silicone rubber. In future contributions, it is aimed to implement the presented thermo-electro-mechanical model within a multi-scale homogenization framework. That allows to analyze multi-physical response of structures used for industrial applications, on multiple scales. Furthermore, it is aimed to validate the computational model using experimental data.

## Figures and Tables

**Table 1:** Parameters for the finite element simulations.

soft matrix
$G_{c}=0.0473$ MPa, $\epsilon_{r}=3.0\;\left[-\right]$, $\alpha =2.83\times 10^{-4}\;K^{-1}$, k=0.15N/(Ks), $\theta_{0}=298\;K$, $\rho_{0}c=1.438\;N/({\mathrm{mm}}^{2}K$).
stiff inclusion
$G_{c}=47.3$ MPa, $\epsilon_{r}=10^{3}\;\left[-\right]$, $\alpha =1.57\times 10^{-5}\;K^{-1}$, k=6.0N/(Ks), $\theta_{0}=298\;K$, $\rho_{0}c=3.190\;N/({\mathrm{mm}}^{2}K$).

## Data Availability

Not applicable.

## Abbreviations

The following abbreviations are used in this manuscript:

EAP	Electro-active polymers
DEA	Dielectric elastomer actuators
MD	Molecular dynamics
